# Gacyclidine improves the survival and reduces motor deficits in a mouse model of amyotrophic lateral sclerosis

**DOI:** 10.3389/fncel.2013.00280

**Published:** 2013-12-27

**Authors:** Yannick N. Gerber, Alain Privat, Florence E. Perrin

**Affiliations:** ^1^Institute for Neurosciences of Montpellier (INM), INSERM U 1051Montpellier, France; ^2^“Integrative Biology of Neurodegeneration,” IKERBASQUE Basque Foundation for Science, Neuroscience Department, University of the Basque CountryBilbao, Spain; ^3^“Integrative Biology of Neuroregeneration,” Faculty of Science, University of Montpellier 2Montpellier, France

**Keywords:** GK11, NMDA receptor antagonist, ALS, survival, locomotion

## Abstract

Amyotrophic lateral sclerosis (ALS) is a fatal neurodegenerative disorder typified by a massive loss of motor neurons with few therapeutic options. The exact cause of neuronal degeneration is unknown but it is now admitted that ALS is a multifactorial disease with several mechanisms involved including glutamate excitotoxicity. More specifically, *N*-methyl-D-aspartate (NMDA)-mediated cell death and impairment of the glutamate-transport has been suggested to play a key role in ALS pathophysiology. Thus, evaluating NMDAR antagonists is of high therapeutic interest. Gacyclidine, also named GK11, is a high affinity non-competitive NMDAR antagonist that may protect against motor neuron death in an ALS context. Moreover, GK11 presents a low intrinsic neurotoxicity and has already been used in two clinical trials for CNS lesions. In the present study, we investigated the influence of chronic administration of two doses of GK11 (0.1 and 1 mg/kg) on the survival and the functional motor activity of hSOD1^G93A^ mice, an animal model of ALS. Treatment started at early symptomatic age (60 days) and was applied bi-weekly until the end stage of the disease. We first confirmed that functional alteration of locomotor activity was evident in the hSOD1^G93A^ transgenic female mice by 60 days of age. A low dose of GK11 improved the survival of the mice by 4.3% and partially preserved body weight. Improved life span was associated with a delay in locomotor function impairment. Conversely, the high dose treatment worsened motor functions. These findings suggest that chronic administration of GK11 beginning at early symptomatic stage may be beneficial for patients with ALS.

## Introduction

Amyotrophic lateral sclerosis (ALS) is a chronic neurodegenerative disease characterized by neuronal death of both lower and upper motoneurons in the spinal cord, the brain stem and the motor cortex. This chronic motor neuronopathy leads to progressive atrophy of skeletal muscles, paralysis and ultimately to death of the patients mainly due to respiratory failure [for review see (Turner et al., 2013)].

Pathogenesis and mechanisms of selective vulnerability of motoneurons in ALS are still largely unknown although, within the past two decades, it has been demonstrated that ALS is a complex multifactorial disease. Indeed many factors including protein misfolding, glutamate-mediated excitotoxicity, oxidative stress and impaired axonal transport may contribute to motoneuron death in ALS [for review see Robberecht and Philips (2013)]. An excessive stimulation of glutamate receptors induces excitotoxic processes; this phenomenon being largely implicated in both acute and chronic neurodegenerative diseases (Olney, 1989; Plaitakis and Constantakakis, 1993; Mehta et al., 2013), and in particular in ALS (Heath and Shaw, 2002; Bogaert et al., 2010). In the objective of translation to clinics, excitotoxicity is one of the key pharmacological targets as attested by the only FDA-approved drug, riluzole (2-amino-6-trifluoromethoxy benzothiazole) which is a modulator of excitatory neurotransmitters activity (including glutamate) (Bogaert et al., 2010). Glutamate interacts with a large range of specific transporters and receptors such as N-methyl-D-aspartate receptors (NMDARs) (Mehta et al., 2013) and a promising, but so far overlooked therapeutic strategy, is to reduce excitotoxicity using NMDA receptor antagonist [for review see Spalloni et al. (2013)]. The low-affinity non-competitive NMDAR antagonist, memantine displayed encouraging results in hSOD1^G93A^ mice (Wang and Zhang, 2005; Joo et al., 2007), an ALS animal model. Clinical trials have shown that memantine is safe and well tolerated by ALS patients (de Carvalho et al., 2010; Levine et al., 2010) but no evidence of its efficacy had been reported yet.

Gacyclidine (GK11), a phencyclidine, is a non-competitive NMDA receptor antagonist, with a selective affinity for NR2B receptors, that had been shown to prevent glutamate-induced neuronal death *in vitro* and is less neurotoxic than other NMDA receptor antagonists (Hirbec et al., 2001; Vandame et al., 2007). Moreover, *in vivo* GK11 exhibits neuroprotective effects following organophosphorous nerve agents-induced convulsions (Bhagat et al., 2005) and following spinal cord injury (Feldblum et al., 2000; Gaviria et al., 2000a,b; Kouyoumdjian et al., 2009; Lonjon et al., 2010).

In this study we have evaluated the effect of chronic treatment of two doses of GK11 on the survival and the locomotor function of hSOD1^G93A^ mice. We demonstrate a dose effect of GK11 on the survival of the mice, treatment with a low GK11 dose induced an increase in life span conversely to a high dose that reduced it. Moreover, treatment-induced increase in survival was associated with a reduction of locomotor function impairment whereas high dose treatment worsened the motor phenotype.

## Materials and methods

### Animals

Transgenic mice carrying the G93A human SOD1 mutation, B6SJL-Tg (SOD1-G93A) 1Gur/J (ALS mice, high copy number) were purchased from The Jackson Laboratory (Bar Harbor, ME, USA) and bred on a B6SJL background. Transgenic mice were identified by PCR and housed in controlled conditions (hygrometry, temperature and 12h light/dark cycle); the environment was not modified over the course of the protocol. Only females were used and litter-matching between groups were done as much as possible. We carried out all animal experiments in accordance with the guidelines approved by the French Ministry of Agriculture and following the European Council directive (2010/63/UE). Every effort was made to minimize the number and suffering of animals. Age of death was defined as functional paralysis of both hindlimbs and a righting reflex >20 s. These criteria follow the commonly accepted guide lines for working on ALS mice (Leitner and Lutz, 2009; Solomon et al., 2011).

### GK11 treatments

Twice a week, mice (transgenic and control) were intra-peritoneally injected with either gacyclidine (two different concentrations; 1 mg/kg or 0.1 mg/kg) (Neuréva, Montpellier, France) or NaCl. The treatment doses were determined according to the following criteria: the dose of 1 mg/kg corresponds to the acute therapeutic dose in rat (Feldblum et al., 2000; Gaviria et al., 2000b) and we wanted to test the possible toxic effect of a chronic administration. Repeated administration of low dose of non-competitive NMDA receptor antagonists induces hyperlocomotion in rat (Wolf and Khansa, 1991; Loscher and Honack, 1992; Matsuoka et al., 2005). Injections were thus not done daily not only to reduce the risk of peritonitis but also to prevent possible interference of injections with behavioral tests. Treatment started at 60 days of age and was carried until the death of the animal. Number of mice: controls injected with GK11 (0.1 mg/Kg, *n* = 10; 1 mg/Kg, *n* = 5) or NaCl (*n* = 14); transgenic mice injected with GK11 (0.1 mg/Kg, *n* = 15; 1 mg/Kg, *n* = 5) or NaCl (*n* = 21).

### Behavioral analysis

#### Catwalk

We used the CatWalk™ (Noldus, Wageningen, The Netherlands) to study dynamic and voluntarily walking patterns of the mice. As previously described (Gerber et al., 2012a) we selected amongst locomotor patterns, the “relative position” that corresponds to the distance between the placement of front and hind paws over one walking step. For data collection, six runs per animal were performed on a weekly basis from day 60 (just prior the first injection) and until animals were not able to correctly cross the walkway due to hindlimb paralysis. For each mouse, a minimum of three runs crossed at the same speed with 3-full step sequence patterns per run were recorded. To accustom the animals to the environment and thus to avoid bias due to stress, we placed transgenic and control littermates mice on the CatWalk 7 and 3 days prior to the first recording session. Catwalk analyses started before the first injection, the next behavioral session was done at least 72 h after treatment. Recordings were performed until the failure to obtain satisfactory paw patterns with the CatWalk analysis system due to hindlimb paralysis and thus absence of paw detection in the transgenic groups. Data analysis was done in collaboration with InnovationNet (Tiranges, France).

#### Open field activity

Spontaneous locomotor activity of mice was monitored in an open field test. Animals were placed in an empty test arena (45 × 45 cm box) and movements automatically recorded. We analyzed the total distance (cm) (Bioseb, Open field, Actitrack software, Vitrolles, France). Recording sessions started at P53 and until the end of life of the transgenic mice; analysis correspond to a weekly 8 min' sessions preceded by 2 min without recordings to avoid any bias due to stress. Open field test were performed on a weekly basis, 48 h after the injection. Recording sessions were performed until mice were unable to move in the test arena

### Statistical analysis

**Figure 1:**
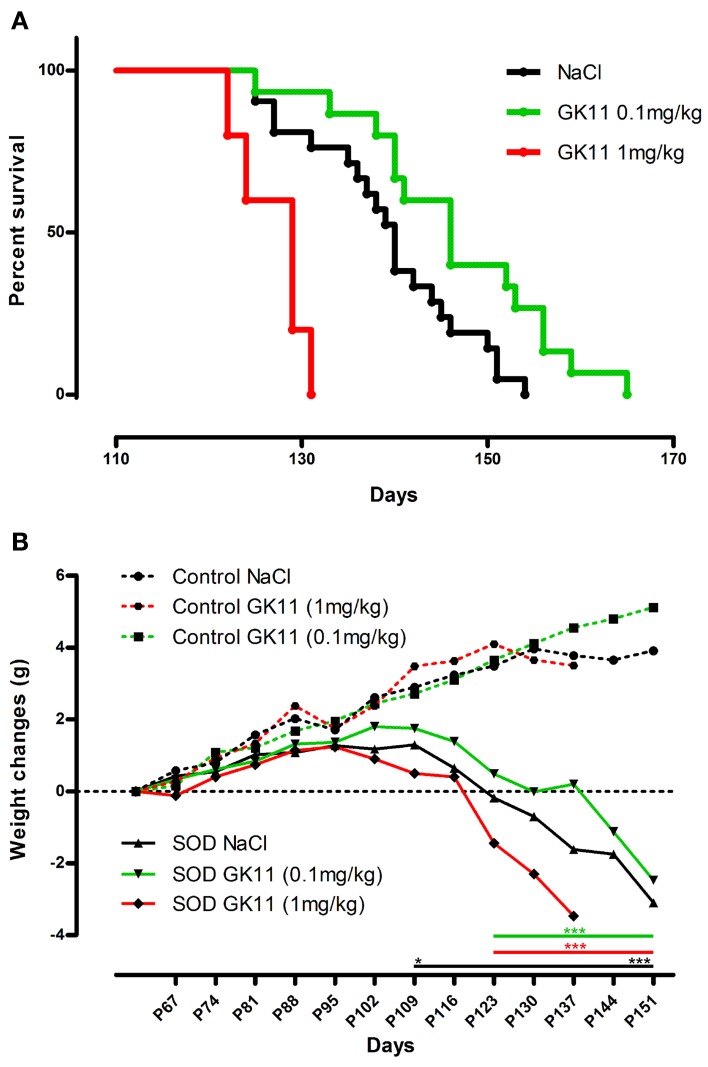
**Dose effect of Gacyclidine on hSOD1^G93A^ mice survival and weight. (A)**—Kaplan-Meier curves of hSOD1^G93A^ mice injected i.p with NaCl (*n* = 21), GK11 0.1 mg/kg (*n* = 15) or GK11 1 mg/kg (*n* = 5). **(B)**—Weight changes (g) in control (dash line) and transgenic mice (continuous line) injected with NaCl, GK11 0.1 mg/kg or GK11.1 mg/kg. Two-ways ANOVA followed by Tukey-Kramer test [*F*_(10, 695) = 96.18_]. Weight of NaCl treated controls and transgenics significantly differs from P109 whereas weight of GK11 (both doses) treated controls and transgenics significantly differ from P123. ^*^p < 0.05 and ^***^p < 0.001.

**Figure 2:**
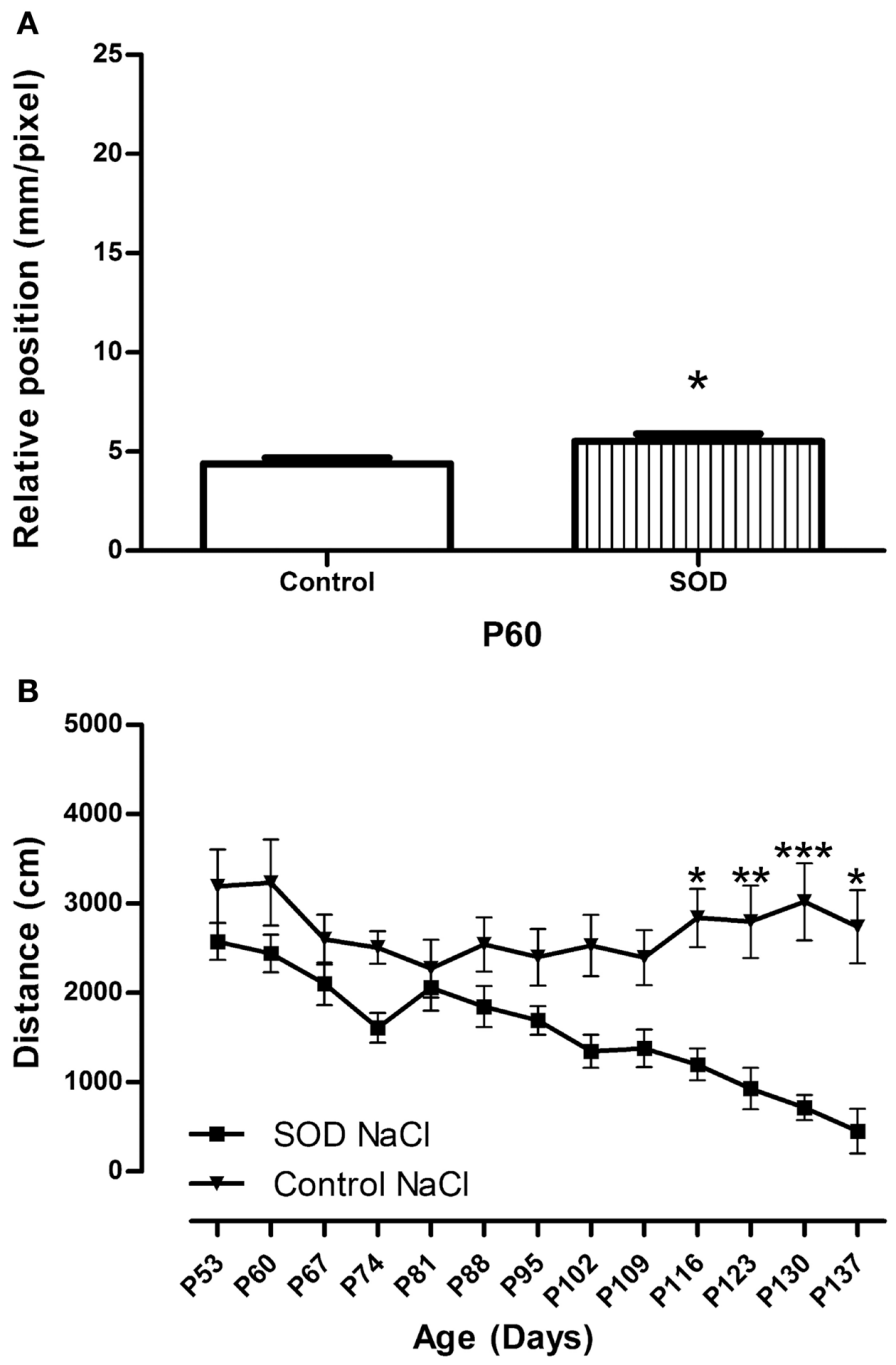
**Locomotor function analysis of hSOD1^G93A^ female mice. (A)**—CatWalk gait analysis. Graph represent the distance between the front and hind paws on a step sequence (“relative position”) of control females (*n* = 32) and hSOD1^G93A^animals (*n* = 41) before treatment. Statistics: *t*-test: ^*^p < 0.05 **(B)**—Open field test. Spontaneous locomotor activity represented by the mean distance covered by control female and hSOD1^G93A^ mice in a weekly 8 min recording test over disease progression. For Open field test a minimum of 10 animals were used from day 53 to 130, then due to the death of hSOD1^G93A^ mice, a minimum of 5 animals were analyzed (P137). Statistics: two ways ANOVA followed by Tukey Kramer test. *F*_(12, 262) = 95.95_. ^*^p < 0.05, ^**^p < 0.01 and ^***^p < 0.001.

**Figure 3:**
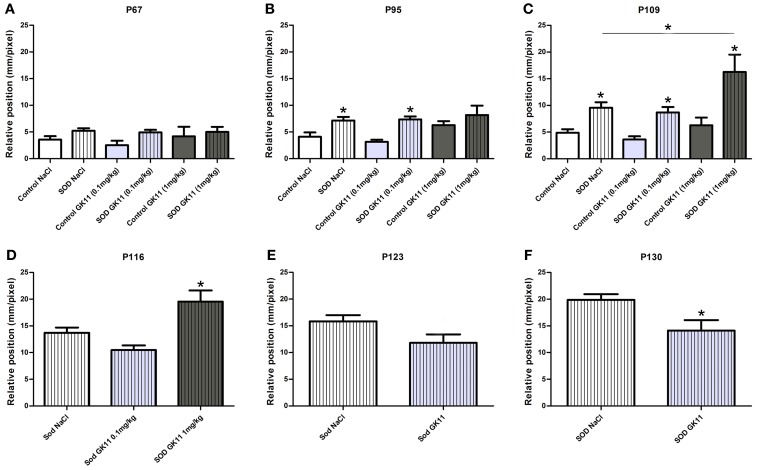
**Evaluation of Gacyclidine effects on hSOD1^G93A^ mice motor pattern: CatWalk gait analysis.** Graphs represent the distance between the front and hind paws on a step sequence (“relative position”). **(A—C)**—control and hSOD1^G93A^ animals (NaCl, GK11 0.1 mg/kg or GK11 1 mg/kg treated)—Respectively at 67, 95, and, 109 days of age. **(D)**—hSOD1^G93A^ animals (NaCl, GK11 0.1 mg/kg or GK11 1 mg/kg injected) at 116 days. **(E, F)** hSOD1^G93A^ animals (NaCl or GK11 0.1 mg/kg injected) at 123 and 130 days. A minimum of 4 and up to 24 animals per time point were analyzed from 67 to 116 days of age; recording were stopped for GK 11 1 mg/kg after P116 due to hindlimb paralysis. Then due to progression of the paralysis a minimum of 8 and up to 15 animals per time point were analyzed from 123 to 130 days of age. Statistics: (**A–C**): two ways ANOVA followed by Tukey Kramer test. **D**: one way ANOVA followed by Tukey Kramer test. (**E**,**F**): one way ANOVA followed by Mann-Whitney test. ^*^p < 0.05.

**Figure 4:**
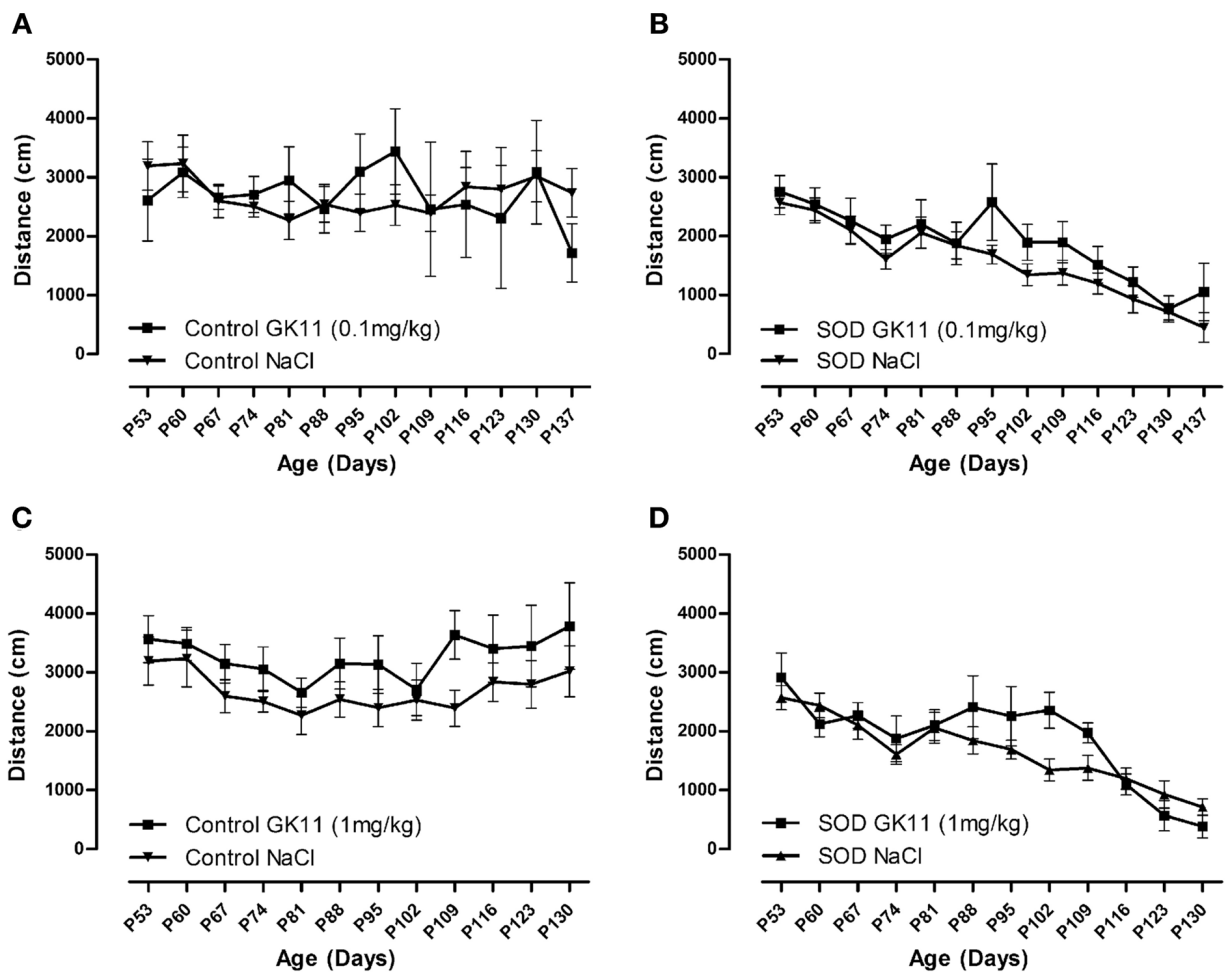
**Evaluation of Gacyclidine effects on the spontaneous motor activity of hSOD1^G93A^ mice: Open Field analysis.** Spontaneous locomotor activity represented by the mean distance covered by female controls and hSOD1^G93A^ mice in a weekly 8 min recording test over disease progression included. **(A**,**B)**—Control and hSOD1^G93A^ mice injected with NaCl or GK11 0.1 mg/kg. **(C**,**D)**—Control and hSOD1^G93A^ mice injected with NaCl or GK11 1 mg/kg. A minimum of 5 animals were analyzed per time point. Statistics: two ways ANOVA followed by Tukey Kramer test.

**Figure A1:**
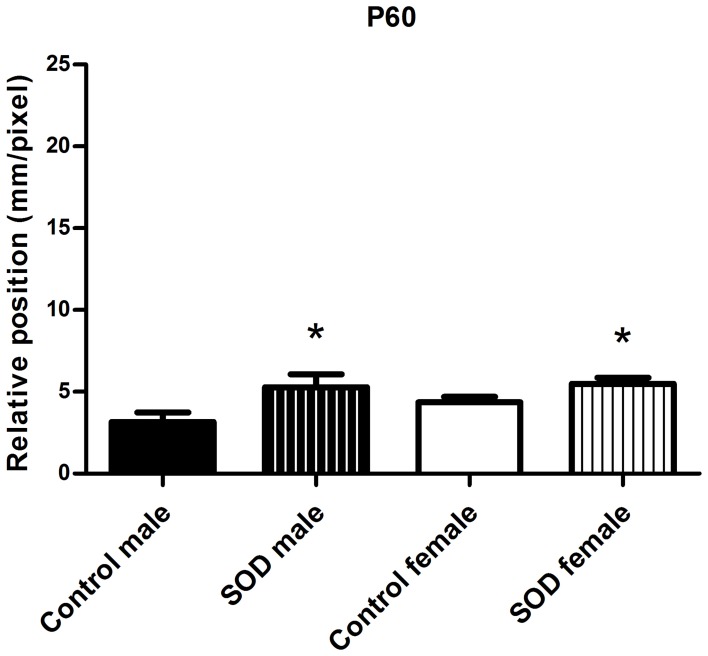
**Locomotor function analysis of hSOD1^G93A^ female and male mice.** CatWalk gait analysis. Graph represent the distance between the front and hind paws on a step sequence (“relative position”) of control males (*n* = 16), hSOD1^G93A^ males (*n* = 11), control females (*n* = 32) and hSOD1^G93A^ animals (*n* = 41) at 60 days of age. Statistics: *t*-test: ^*^p < 0.05.

Kaplan Meier analysis and log-rank test were applied for survival curves (Figure 1A). Two ways ANOVA followed by Tukey-Kramer test was used for all analysis that consist on a combination of a treatment (at three different doses, NaCl, GK11 0.1 mg/kg and GK11 1 mg/kg) on two distinct genotypes over time (Figures 1B, 2B, 3A–C, 4). From 116 days onward transgenic and control mice could not be directly compared due to speed differences, moreover due to death GK11 1 mg/kg-treated transgenic mice were not included from P123 onwards. We thus used one way ANOVA followed by Tukey-Kramer test for Figure 3D and one way ANOVA followed by Mann-Whitney test for Figures 3E,F. For all analysis at P60 i.e., before the treatment started *t*-test was done (Figures 1A, A1). CatWalk data consist on replicates of a minimum of 3 and a maximum of 6 runs (same speed with 3 full step sequence) per animal in all cases these values were averaged. Experiments were designed to reach a 95% power to detect a 10% or greater difference between groups. We used GraphPad Prism version 5.03 (GraphPad software, CA, USA, and Minitab 15, Minitab Inc, USA).

## Results

### Dose effect of gacyclidine on the survival of the mice

We first assessed the influence of a chronic treatment with GK11 (0.1 mg/kg), a non-competitive NMDA receptor antagonist, on the survival of hSOD^G93A^ female mice. Animals treated with GK11 display an increased survival as compared to animals injected with NaCl. Median survivals are indeed of 146 and 140 for mice injected with GK11 and NaCl respectively; this represent a significant 4.3% increase in life span (Kaplan Meier, *P*-value = 0.0341) (Figure 1A).

To evaluate the possible toxicity margin of the molecule, we injected mice with a 1 log higher GK11 concentration (1 mg/kg). Mice chronically injected with this high dose show a significant decrease in lifespan as compared to the littermates injected with NaCl. Median survivals are indeed of 129 and 140 for mice injected with GK11 and NaCl respectively; this represents a significant 7.9% decrease in life span (Kaplan Meier, *P*-value = 0.006) (Figure 1A).

Over the progression of the disease, transgenic mice lost weight due to muscular atrophy. We monitored body weight changes from P60 until death. NaCl injected transgenic mice weight differs significantly from those of the NaCl injected control group from P109 whereas weight of GK11 treated transgenic mice (both doses) is significantly different from GK11 treated control mice from P123 (Figure 1B). There is no effect of GK11 treatment on the body weight of control mice.

We thus evidence a beneficial effect of a chronic low dose of GK11 on the survival of hSOD1^G93A^ female mice whereas a high dose of GK11 has a detrimental effect.

### Locomotor function is altered at 60 days of age in female hSOD1^G93A^ mice

In the perspective of translational studies, it is important to apply the therapeutic strategy at early symptomatic period. Experiments were done on female mice, it was thus mandatory to thoroughly characterize disease onset and progression in female hSOD1^G93A^ mice. We have used the previously described combination of CatWalk and open field analysis (Gerber et al., 2012a) to evaluate locomotor alterations. To compare with our former motor analysis on male hSOD1^G93A^ mice, we tested the female mice on a weekly basis from 60 days of age and selected the same parameters. We quantified the spontaneous motor activity using the open field system and the “relative position” of the paws (distance between front and hind footprints over a step cycle) using the CatWalk system.

As a prerequisite for accurate CatWalk investigation, we verified if the B6SJL strain is suitable for gait analysis. In that aim, we analyzed the stride of control females from P60 to P130. It had indeed been shown that stride must remain stable in control mice all along the study protocol to avoid any misleading conclusions (Clarke and Still, 1999). The stride remains identical throughout our study (Table A1).

At 60 days of age hSOD1^G93A^ female mice present an alteration in gait pattern appearing as the inability to draw their hind limbs up to the previous position of their front limbs (Figure 2A) This alteration increased further up to 109 days of age (Figures 3A–C, SOD NaCl vs. control NaCl). To evaluate the spontaneous motor activity of hSOD1^G93A^ and control mice, we used the open field system. We evidence a decreased in the mean distance covered by transgenic animals as compared to their control littermate from P116 (Figure 2B).

Combination of CatWalk and open field analysis allows an accurate description of motor alterations and permit to identify gait pattern alteration, and thus clinical onset of the disease, at 60 days of age. This is in agreement with our previous results in male hSOD1^G93A^ mice (Gerber et al., 2012a) (Figure A1).

### Increased life span correlated with delayed motor deficits

We then used the CatWalk system to tentatively correlate the effects of the drug on the lifespan with modification of disease onset and/or progression of the disease; we compared locomotor functions of transgenic hSOD1^G93A^ females injected with either GK11 (0.1 mg/kg or 1 mg/kg) or NaCl. In parallel, we evaluated whether GK11 had an effect on non-transgenic animal by comparing treated versus NaCl injected control mice.

From 67 days to 109 days, the relative position is identical in transgenic groups injected with GK11 0.1 mg/kg or 1 mg/kg compared to transgenic mice injected with NaCl. Controls animals of all groups (GK11 0.1 mg/kg, 1 mg/kg or NaCl) remain stable over the same period. However, transgenic mice (0.1 mg/kg and NaCl) show an alteration of the motor pattern when compared to their respective controls that increased from 95 to 109 days of age. At 109 days, transgenic mice injected with GK11 at high concentration show a critically altered phenotype compared not only to controls but also to the transgenic group injected with NaCl (Figures 3A–C). From 116 days onward, due to hindlimb paralysis, transgenic and control mice do not achieve the CatWalk test at the same speed and thus, referring to Clarke's et al. study (Clarke and Still, 1999), cannot be compared. We thus analyzed separately hSOD1^G93A^ and control mice. At 116 days, mice injected with GK11 1 mg/kg show an altered phenotype compared to the NaCl injected mice (Figure 3D). No satisfactory recording sessions were obtained for the GK11 1 mg/kg from P123 onwards (Figures 3E,F). GK11 0.1 mg/kg treated mice exhibit a reduction of locomotor function impairment compared to the transgenic NaCl group at P130 (Figure 3F). No difference between control groups were seen over the same period (data not shown). These results suggest that treatment with a low dose of GK11 induces a slight but significant delay in hindlimb paralysis progression. Oppositely, treatment with a high dose of GK11 worsened the motor pattern.

We used an open field system to measure the spontaneous locomotor activity of control and transgenic mice. No difference has been noted in the mean crossed distances when we compared both GK11 doses (0.1 and 1 mg/kg) and NaCl injected mice for both control and transgenic groups (Figures 4A–D).

## Discussion

Glutamate mediated excitotoxicity is involved in ALS pathogenesis (Foran and Trotti, 2009) and several studies have evaluated the pharmacological profile of a number of molecules acting on glutamate release and transport [for review (Corona et al., 2007; Bogaert et al., 2010)]. Gacyclidine (GK11), a non-competitive NMDA receptor antagonist, has already been used in the clinics (Hirbec et al., 2001; Tadie et al., 2003; Lepeintre et al., 2004) and a single GK11 dose of 1 mg/kg has demonstrated neuroprotective effects on a model of photochemical spinal cord injury (Gaviria et al., 2000b). Chronic GK11 administration has never been reported. In view of these data, we decided to analyze the impact of a chronic treatment of GK11 on the survival and locomotor activity of hSOD1^G93A^ mice.

Our study clearly shows a dose response effect; indeed although a high GK11 dose is detrimental by shortening the life span of the mice by 7.9%, a low dose induces a 4.3% increase in survival vs. controls. Moreover, this increased life span is associated with a reduction in walking pattern deficits whereas high dose treatment worsened the motor phenotype.

### Gender effects on survival and functional deficits in the SOD1^G93A^ mouse model of ALS

We have recently carried out a thorough analysis analyses of survival and motor function in hSOD1^G93A^ male mice (Gerber et al., 2012a,b). For the present study we have used females. Transgenic females survive significantly longer than males; median survivals were of 129 and 140 days for males and females respectively. Comparison with a previous study (Heiman-Patterson et al., 2005) attests for the stability of this mouse model; indeed reported life span was of 128 and 133 days for males and females respectively.

We have evaluated locomotor function of female hSOD1^G93A^ mice using the same combination of dynamic walking patterns and spontaneous motor activity analysis as previously done with males (Gerber et al., 2012a). As for males, we detect early functional deficits that confirm symptoms onset at 60 days of age, i.e., 20 days earlier than previously described (Figure A1). Indeed, dynamic gait analysis using the CatWalk system demonstrates alteration in gait pattern for both males and females from 60 days onwards. Spontaneous motor activity evaluated with an open field highlighted a decrease in the mean distance covered by transgenic animals as compared to their control littermate from 116 days onwards for females as compared to 102 days of age onwards for males (Gerber et al., 2012a). This difference may reflect gender-specific mechanism of synaptic impairment since a recent study demonstrated sexual dimorphism in the decline of the presynaptic machinery at an early symptomatic stage (Naumenko et al., 2011). Mean survival of hSOD1^G93A^ mice is of 140 days for female as compared to 129 days for males (Gerber et al., 2012a), however, even if female mice survive longer than male counterparts, early motor symptoms impairment are detected at 60 days of age in both gender.

### Antiglutamate drugs

One of the popular hypothesis regarding mechanisms involved in ALS is that an excessive activation of glutamate receptors may be responsible for motoneuron death (Corona et al., 2007). NMDA receptors are known for their permeability to cations and in particular to calcium; their over activation leading to excitoxic phenomenon. Recent findings have shown that subtypes of AMPA receptors, that either do not express the GluR2 subunit or express an edited form of this subunit, present a similar permeability to calcium than NMDA receptors (Van Den Bosch et al., 2000; Kawahara et al., 2006; Corona et al., 2007). These AMPA receptor subtypes are widely expressed in motoneurons and are therefore involved in excitotoxic processes (Carriedo et al., 1996; Gerardo-Nava et al., 2013). However, modulating AMPA excitoxicity mediated pathway is mostly ineffective in clinical trials and induce various side effect (such as dizziness and Ataxia) [for review see (Chang et al., 2012)]. Nevertheless, perampanel, a non-competitive AMPA receptor antagonist to AMPAR is very promising in the treatment of epilepsy (Rogawski, 2011). Several antiglutamate drugs have been evaluated in ALS [for review see (Gibson and Bromberg, 2012)]. Riluzole is the only approved treatment and prolongs median survival by about 2 to 3 months in ALS patients (Miller et al., 2012) and by 10.5% in mice (Gurney et al., 1996). Riluzole induces an inhibition of glutamate release and modulation of both alpha-amino-3-hydroxy-5-methyl-4-isoxazolepropionic acid (AMPA) and N-methyl-d-aspartate (NMDA) receptors (Jin et al., 2010; Cifra et al., 2013). However, Riluzole displays other diverse and multiple non-specific pharmacological properties (Cheah et al., 2010) that may cause adverse effects such as nausea, asthenia and raised alanine transferase (Miller et al., 2012). As recently pinpointed (Spalloni et al., 2013) the role of NMDA receptors activation in ALS has not received much attention, even if a low affinity non-competitive NMDAR antagonist, memantine, has shown promising results in an ALS animal model. In this context, evaluations of new therapeutic agents are of utmost importance. GK11, a high affinity uncompetitive NMDAR antagonist, presents a low intrinsic neurotoxicity, paucity of side effects attributed to its selectivity for NR2 B containing receptors (Vandame et al., 2007). No interaction was found with glutamatergic AMPA, kainate, or σ1 and σ2 receptors (Hirbec et al., 2001).

In our study, early symptomatic (starting at P60) bi-weekly chronic administration of a low dose of GK11 (0.1 mg/kg) yields a 4.3% increase in the life span of the female hSOD1^G93A^ mice. These results are similar to the effects of memantine (Wang and Zhang, 2005; Joo et al., 2007). Conversely to memantine (Joo et al., 2007), increase in survival induced by GK11 is associated with a significant protective effect on locomotor deterioration at the end stage of the disease. Of particular interest is the fact that, to-date, GK11 is the most specific NMDA receptor antagonist efficient in an animal model of ALS, thus definitely pinpointing the involvement of this receptor in the pathogenesis of the disease. High dose GK11 treatment leads to a decrease in the life span of female hSOD1^G93A^ associated to a more abrupt development of motor symptoms and hindlimb paralysis. Importantly, high dose drug treatment has no effect on weight loss, CatWalk or open field performances in the control group as reported in a recent study in rats undergoing an acute treatment (Vandame et al., 2013). This toxic side effect might reflect an induction of specific cascade events in the SOD context.

In conclusion, we demonstrate here that a chronic administration (starting at early symptomatic stage) of a non-competitive NMDA receptors antagonist significantly increases the survival of a mouse model of ALS. Indeed, a chronic low dose of GK11 prolongs the life span and reduces weight loss and motor deficits in hSOD1^G93A^ female mice. These results, combined with those from previous studies on CNS injury, demonstrating in patients a relative efficacy and the absence of side effects (Tadie et al., 2003; Lepeintre et al., 2004), suggest that low dose of GK11 could be used as a new and effective NMDA receptor antagonist for ALS patients.

## Author contributions

Yannick N. Gerber: study conception and design, acquisition of data, analysis and interpretation of data and drafting of manuscript. Alain Privat: study conception and design and critical revision. Florence E. Perrin: study conception and design, analysis and interpretation of data and critical revision.

## Conflict of interest statement

The authors declare that the research was conducted in the absence of any commercial or financial relationships that could be construed as a potential conflict of interest.

## Appendix

**Table A1:** **Frontlimb and hindlimb stride (mm) of control mice at P60 (*n* = 22), Control NaCl at P81 (*n* = 12), P109 (*n* = 13) and P130 (*n* = 11)**.

**Colonne1**	**P60**	**P81**	**P109**	**P130**
Frontlimb (mm)	58.81 ± 1.275	59.00 ± 1.317	59.14 ± 1.549	61.48 ± 2.053
Hindlimb (mm)	59.28 ± 1.393	59.27 ± 1.372	59.14 ± 1.507	61.20 ± 1.945
